# Supplementary material for: Predicting genes associated with RNA methylation pathways using machine learning
Source: Commun Biol. 2022 Aug 25;5:868. doi: 10.1038/s42003-022-03821-y (PMC9411552; doi:10.1038/s42003-022-03821-y)
Supplement: Supplementary file 1 — Supplementary Information [file 42003_2022_3821_MOESM1_ESM.pdf]

a) b)

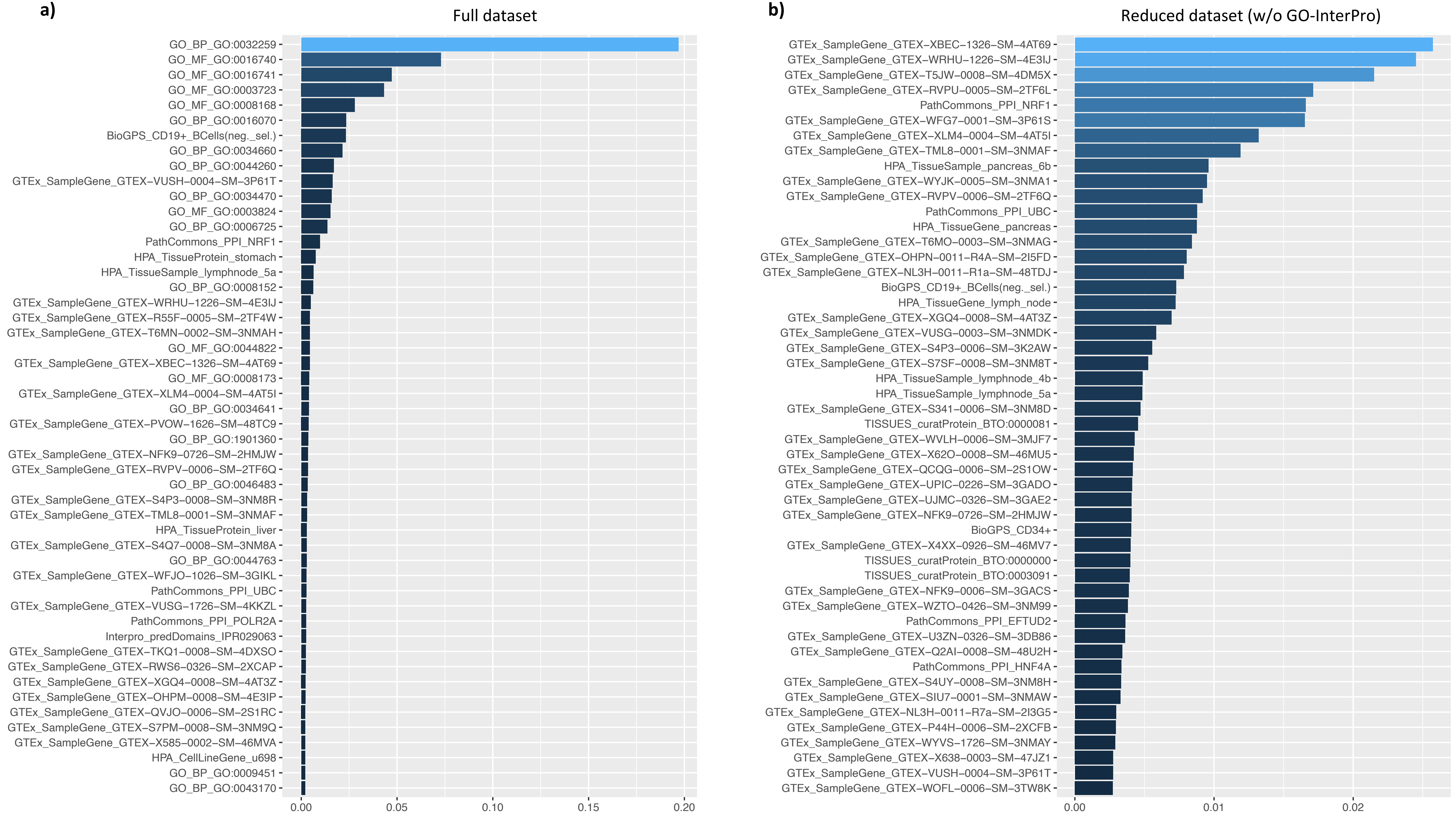

Supplementary Figure 1. Feature importance. Top 50 most informative features ranked by their relative importance in predictive modelling based on the **a)** full and **b)** reduced feature sets.

Top 1% predictions overlap (quantile > 0.99)

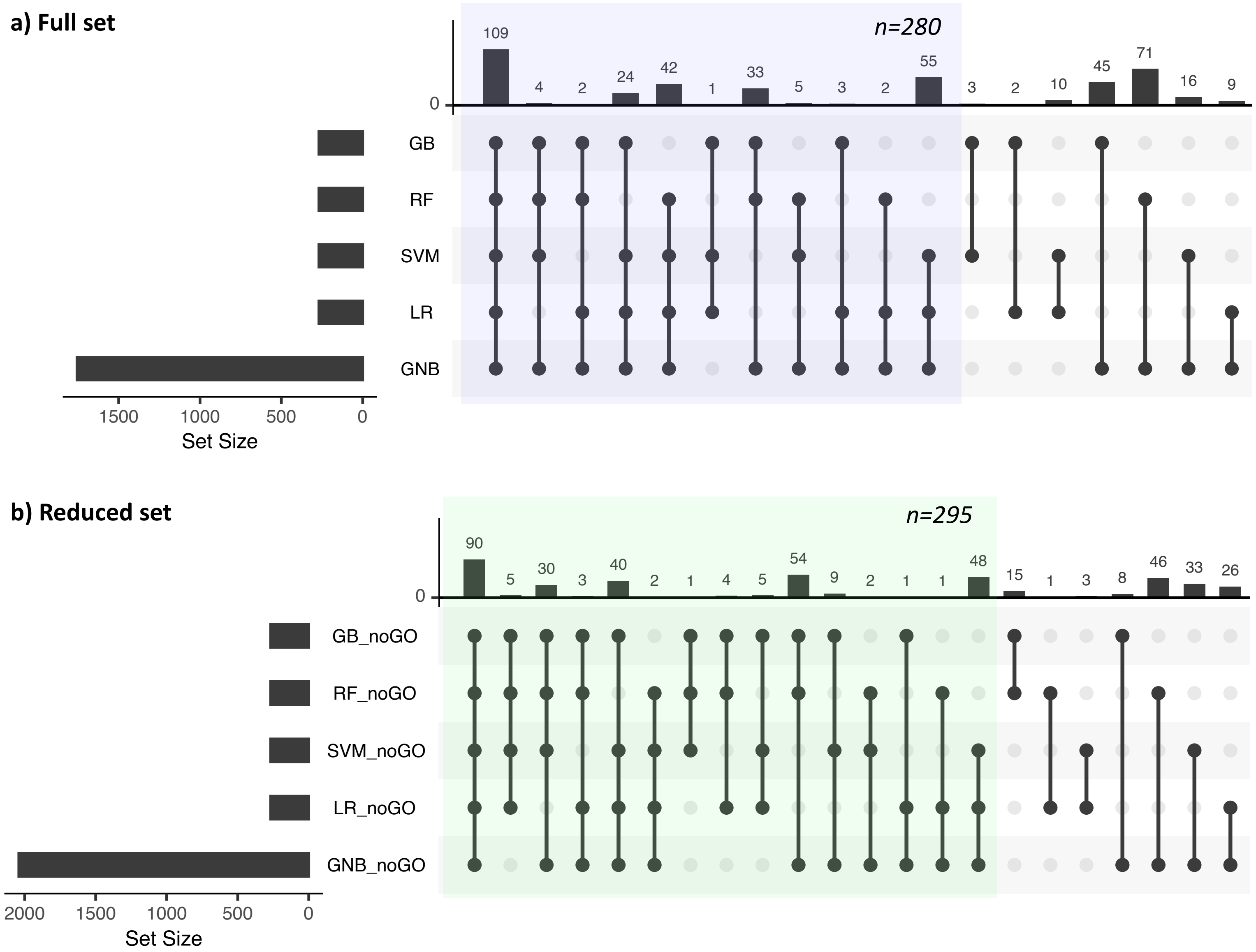

Supplementary Figure 2. High-confidence prediction overlap across models. Overlap of genes in the top 1% of the probability distribution for Class 1 for each model ensemble trained on the **a)** full and **b)** reduced feature sets.

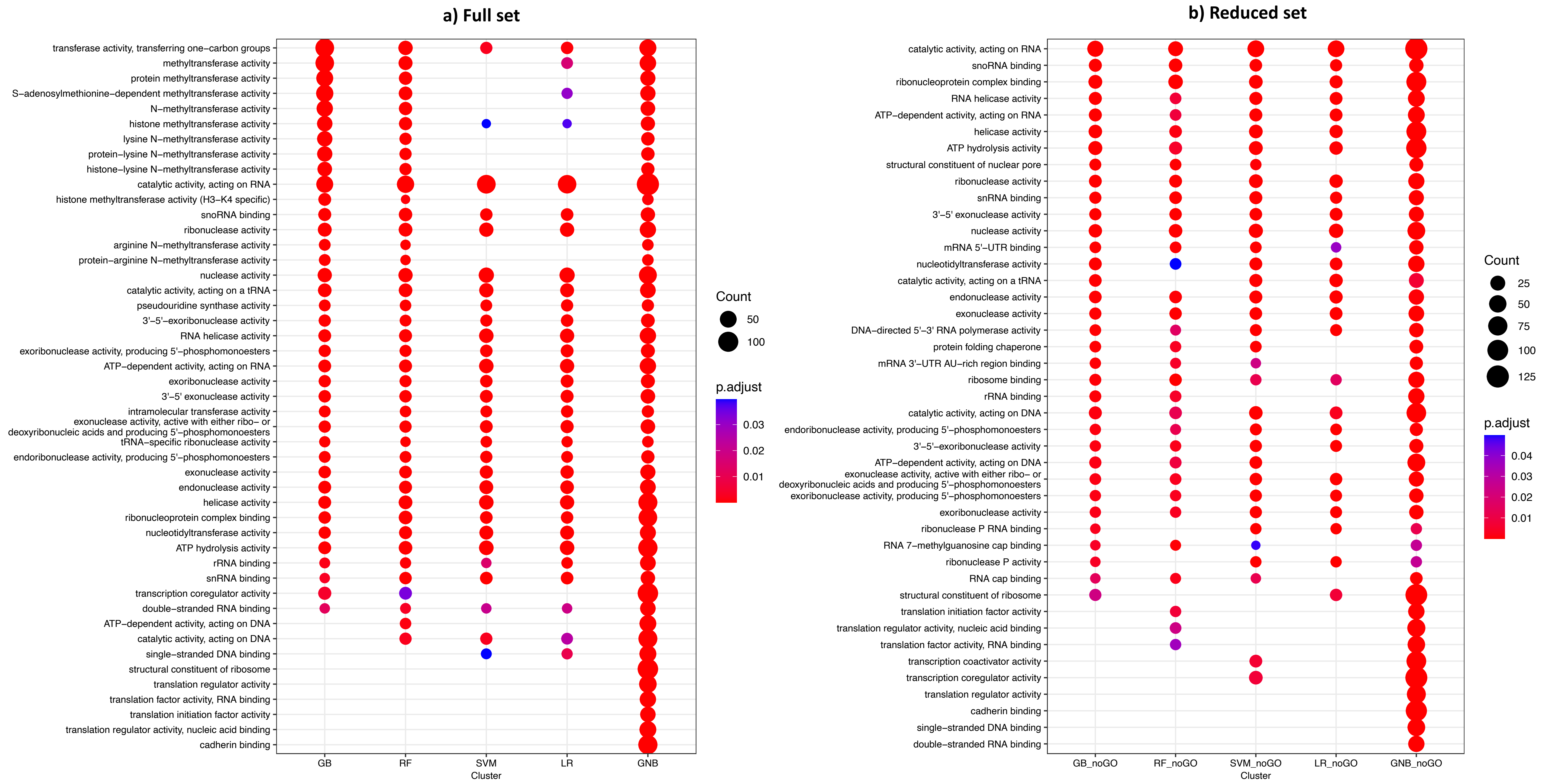

Supplementary Figure 3. Functional enrichment analyses of high-confidence predictions. GO enrichment analysis of genes in the top 1% of the probability distribution for Class 1 for each model ensemble trained on the **a)** full and **b)** reduced feature sets.

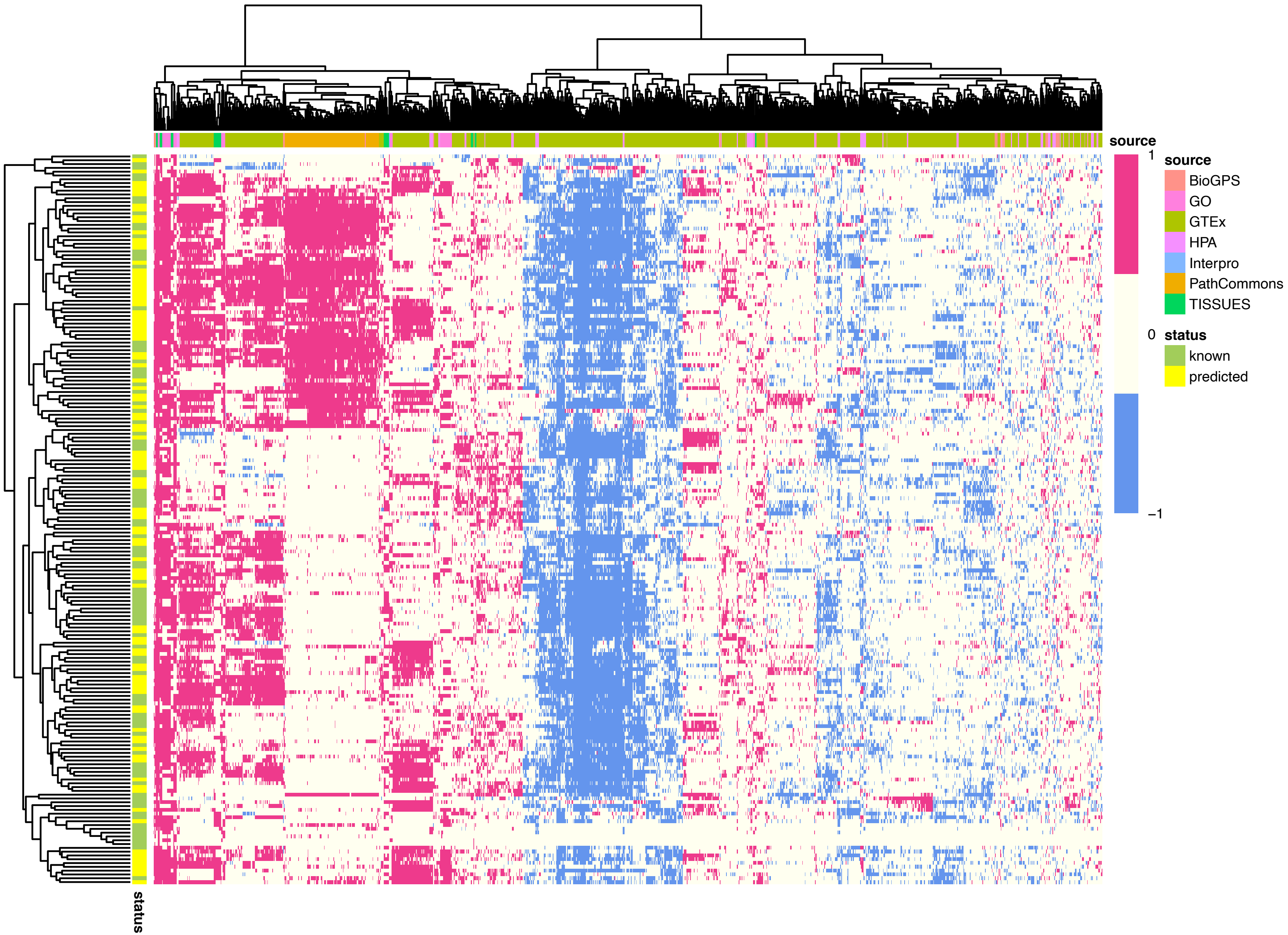

Supplementary Figure 4. Heatmap of predicted and known RNA methylation genes. Hierarchical clustering analysis of predicted plus positive genes shows no evident split between predictions (yellow) and known RNA methylation genes (green). Features (columns) used for machine learning are shown in different colours based on the data source.

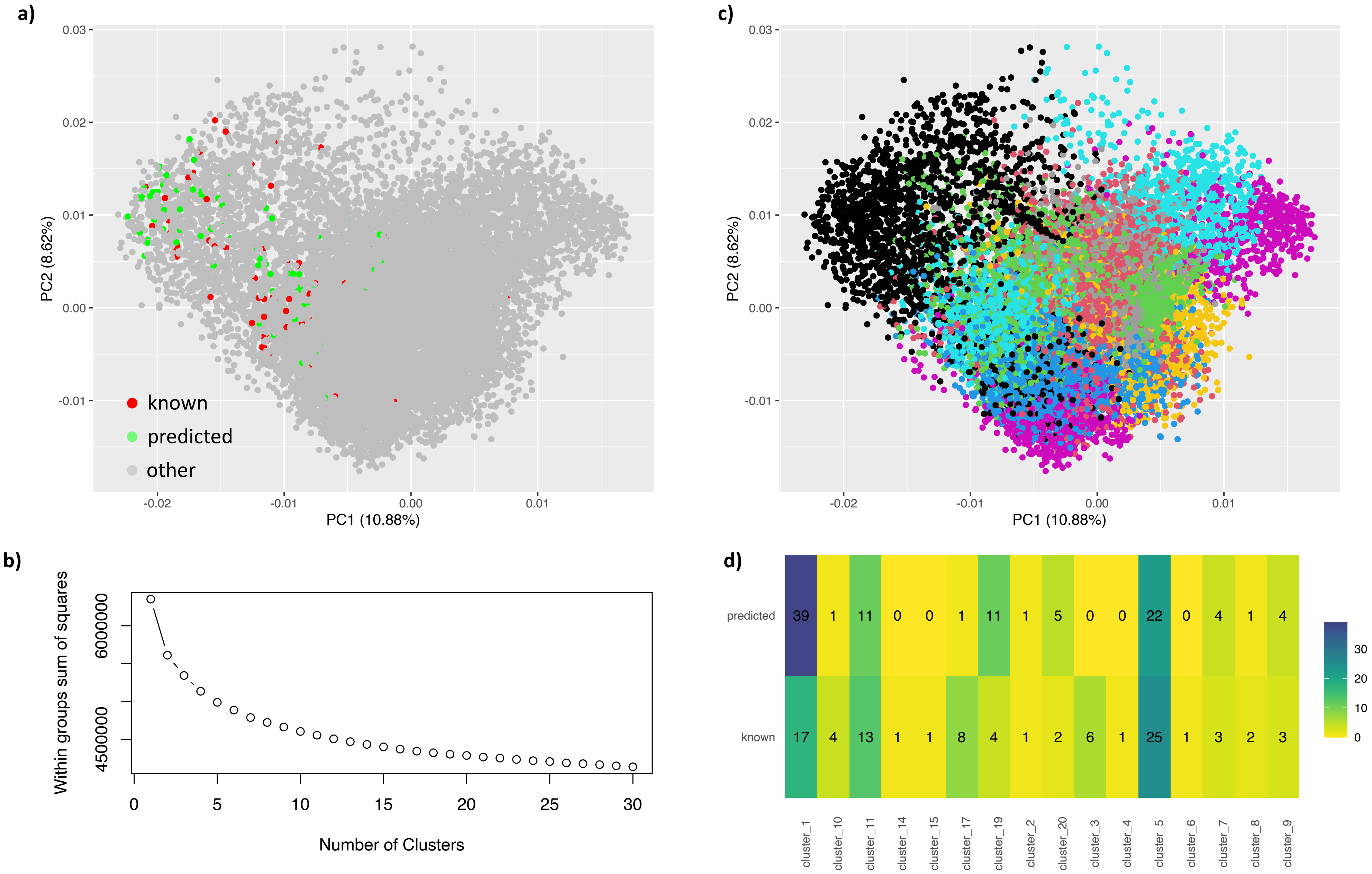

Supplementary Figure 5. Unsupervised clustering analysis based on the same feature set used in supervised learning. a) Principal Component Analysis (PCA) of all human genes based on the selected 1,505 features used in our machine learning models. Previously known RNA methylation genes are marked in red, whereas novel predictions in green; **b)** Elbow plot for selecting the optimal number of clusters (k) for a k-means clustering analysis; **c)** PCA plot with different colours highlighting the inferred k-means clusters; **d)** Number of previously known RNA methylation genes and new predictions across the inferred clusters.

GB predicted probability score – test data combined

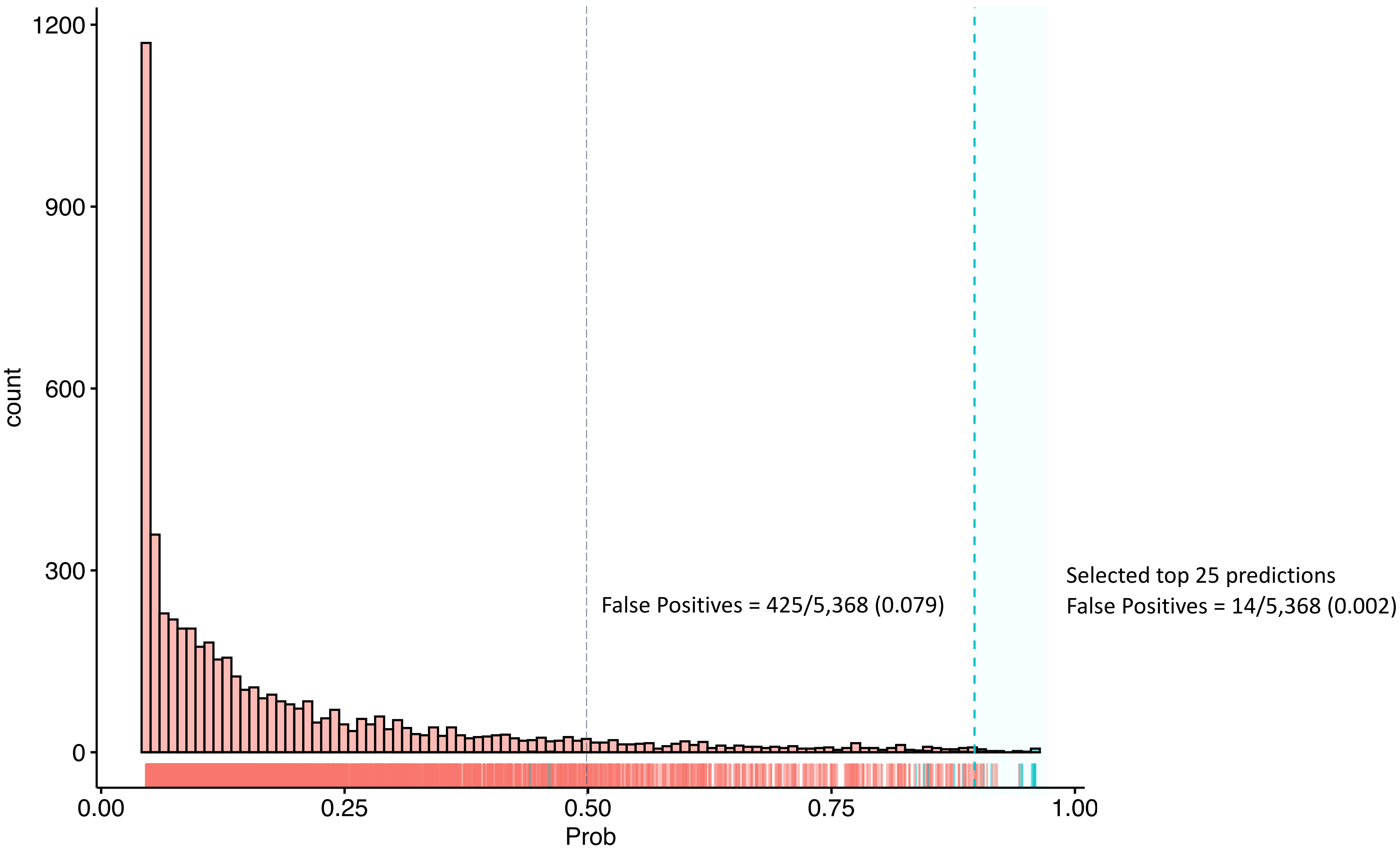

Supplementary Figure 6. Histogram of predicted probability score (Class 1) across all test data combined. The estimated False Positive Rate of the GB model ensemble based on the combined set of hold-out data was 0.079. Considering the top 25 predictions only, the False Positive Rate was 0.002.
