# Supplementary material for: Predicting genes associated with RNA methylation pathways using machine learning
Source: Commun Biol. 2022 Aug 25;5:868. doi: 10.1038/s42003-022-03821-y (PMC9411552; doi:10.1038/s42003-022-03821-y)
Supplement: Supplementary file 2 — Description of Additional Supplementary Data [file 42003_2022_3821_MOESM2_ESM.pdf]

Description of Additional Supplementary Files

File name: Supplementary Data 1
Description: Known RNA methyltransferases and related proteins used as positive set (Class 1).

File name: Supplementary Data 2
Description: Gene-feature omics datasets used in machine learning analyses (source Harmonizome).

File name: Supplementary Data 3
Description: Highly informative features based on models trained on the reduced feature set, and their frequency in the top 100 features across all models of the classifier ensemble.

File name: Supplementary Data 4
Description: Top 100 gene predictions based on the GB model ensemble of the full feature set.

File name: Supplementary Data 5
Description: Predicted probability score across all models and Personalised PageRank score of top 100 model predictions based on PPI data (source: STRING).
