# Supplementary material for: Predicting genes associated with RNA methylation pathways using machine learning
Source: Commun Biol. 2022 Aug 25;5:868. doi: 10.1038/s42003-022-03821-y (PMC9411552; doi:10.1038/s42003-022-03821-y)
Supplement: Supplementary file 4 — Reporting summary [file 42003_2022_3821_MOESM4_ESM.pdf]

nature portfolio

Corresponding author(s): Georgia Tsagkogeorga, Hendrik Weisser, Namshik Han

Last updated by author(s): Jul 4, 2022

Reporting Summary

Nature Portfolio wishes to improve the reproducibility of the work that we publish. This form provides structure for consistency and transparency in reporting. For further information on Nature Portfolio policies, see our Editorial Policies and the Editorial Policy Checklist.

Statistics

For all statistical analyses, confirm that the following items are present in the figure legend, table legend, main text, or Methods section.

n/a	Confirmed	
☐	☒	The exact sample size (*n*) for each experimental group/condition, given as a discrete number and unit of measurement
☒	☐	A statement on whether measurements were taken from distinct samples or whether the same sample was measured repeatedly
☐	☒	The statistical test(s) used AND whether they are one- or two-sided<br>*Only common tests should be described solely by name; describe more complex techniques in the Methods section.*
☒	☐	A description of all covariates tested
☐	☒	A description of any assumptions or corrections, such as tests of normality and adjustment for multiple comparisons
☒	☐	A full description of the statistical parameters including central tendency (e.g. means) or other basic estimates (e.g. regression coefficient) AND variation (e.g. standard deviation) or associated estimates of uncertainty (e.g. confidence intervals)
☒	☐	For null hypothesis testing, the test statistic (e.g. *F*, *t*, *r*) with confidence intervals, effect sizes, degrees of freedom and *P* value noted<br>*Give P values as exact values whenever suitable.*
☒	☐	For Bayesian analysis, information on the choice of priors and Markov chain Monte Carlo settings
☐	☒	For hierarchical and complex designs, identification of the appropriate level for tests and full reporting of outcomes
☒	☐	Estimates of effect sizes (e.g. Cohen's *d*, Pearson's *r*), indicating how they were calculated

Our web collection on statistics for biologists contains articles on many of the points above.

Software and code

Policy information about availability of computer code

Data collection	No software used. Data were downloaded from https://maayanlab.cloud/Harmonizome/
Data analysis	For the machine learning analyses, we developed custom code publicly available at GitHub https://github.com/storm-therapeutics/ML_RNA_methylation. All meta-analyses were performed using the R software environment (v. 4.0.5).

For manuscripts utilizing custom algorithms or software that are central to the research but not yet described in published literature, software must be made available to editors and reviewers. We strongly encourage code deposition in a community repository (e.g. GitHub). See the Nature Portfolio guidelines for submitting code & software for further information.

Data

Policy information about availability of data

All manuscripts must include a data availability statement. This statement should provide the following information, where applicable:

- Accession codes, unique identifiers, or web links for publicly available datasets
- A description of any restrictions on data availability
- For clinical datasets or third party data, please ensure that the statement adheres to our policy

The Python scripts used for the machine learning analyses are available at GitHub https://github.com/storm-therapeutics/ML_RNA_methylation.

Field-specific reporting

Please select the one below that is the best fit for your research. If you are not sure, read the appropriate sections before making your selection.

☒ Life sciences ☐ Behavioural & social sciences ☐ Ecological, evolutionary & environmental sciences

For a reference copy of the document with all sections, see nature.com/documents/nr-reporting-summary-flat.pdf

Life sciences study design

All studies must disclose on these points even when the disclosure is negative.

Sample size	No sample size calculation was performed. For the machine learning analyses, we sampled all known RNA methylation genes as our positive class. These were either methyl-writers (known RNA methyltransferases and their partner proteins in protein complexes), or enzymes previously annotated as putative RNA methyltransferases.The remaining genes of the human genome were divided into sets of equal size to the positive samples, and used as negative samples.
Data exclusions	No data were excluded.
Replication	To ensure reproducibility, we made our code publicly available at GitHub. We also performed our analyses from a fixed random seed.
Randomization	Randomization was used in our negative class sampling. We assembled multiple stratified training and test datasets by randomly sampling a number of genes equal to our positive set from the remaining genome, ensuring that all genes of our initial dataset were sampled exactly once.
Blinding	Not applicable.

Reporting for specific materials, systems and methods

We require information from authors about some types of materials, experimental systems and methods used in many studies. Here, indicate whether each material, system or method listed is relevant to your study. If you are not sure if a list item applies to your research, read the appropriate section before selecting a response.

Materials & experimental systems

n/a	Involved in the study
☒	☐ Antibodies
☒	☐ Eukaryotic cell lines
☒	☐ Palaeontology and archaeology
☒	☐ Animals and other organisms
☒	☐ Human research participants
☒	☐ Clinical data
☒	☐ Dual use research of concern

Methods

n/a	Involved in the study
☒	☐ ChIP-seq
☒	☐ Flow cytometry
☒	☐ MRI-based neuroimaging
